# Continuous time series analysis on the effects of induced running fatigue on leg symmetry using kinematics and kinetic variables: Implications for knee joint injury during a countermovement jump

**DOI:** 10.3389/fphys.2022.877394

**Published:** 2022-08-17

**Authors:** Zixiang Gao, Liang Zhao, Gusztáv Fekete, Gábor Katona, Julien S. Baker, Yaodong Gu

**Affiliations:** ^1^ Faculty of Sports Science, Ningbo University, Ningbo, China; ^2^ Faculty of Engineering, University of Pannonia, Veszprém, Hungary; ^3^ Savaria Institute of Technology, Eötvös Loránd University, Budapest, Hungary; ^4^ Hungarian University of Agriculture and Life Sciences, Gödöllő, Hungary; ^5^ Department of Sport and Physical Education, Hong Kong Baptist University, Hong Kong, China

**Keywords:** symmetry function, running-induced fatigue, countermovement jump, statistical parameter mapping, landing

## Abstract

This study investigates the symmetry change in joint angle and joint moment of knee joints following a Running-Induced Fatigue counter movement Jump. Twelve amateur runners volunteered to participate in the study. A prolonged running protocol was used to induce fatigue. Joint angle and moment were recorded during the push and flexion phase of the CMJ before and immediately after fatigue. Borg scale (RPE>17) and real-time heart rate monitoring (HR>90%HRmax) were used to confirm running fatigue. Symmetry function (SF) was used to assess the symmetry of the knee Angle and moment variation parameters over the entire push-off and landing phases based on time series analysis. Paired sample *t*-test was used to examine changes in SF before and after acute fatigue. The Angle and moment of the knee are asymmetrical in all planes (SF > 0.05), with SF ranging from 5 to 130% in angle and 5–110% in moment. There was a significant increase in knee joint angle asymmetry in the horizontal plane during the push-off and landing stage following the prolonged - Running Protocol implementation. These increases in asymmetry are mainly caused by excessive external rotation of the dominant knee joint. These findings indicate that fatigue-induced changes during CMJ may progress knee movement pattern asymmetry in the horizontal plane.

## Introduction

Countermovement jumping (CMJ) is commonly used to assess leg strength under the slow stretch shortening cycle (SSC) and low stretch load conditions (Maulder and Cronin, 2005). The CMJ is a key component used frequently in bounding sports. Previous studies reported that the relationship between leg strength and functional performance (e.g., running and sprinting) have generally used bilateral vertical jumping or its derivatives as assessment methods (Nesser et al., 1996). CMJ places a relatively high external load on the lower extremities during landing compared to tasks that require less movement, such as jogging. Vertical ground reaction forces (GRF) can reach 3 to 3.5 times individual body weight (Schmitt and Rudolph, 2007), far more than the 2 to 2.5 BW forces experienced during jogging (Farley et al., 1998). The knee joint contributes about 49% of the total positive work during CMJ (Hubley and Wells, 1983). As a significant contributor to CMJ, the incidence of knee joint injury should also be a concern for athletes and coaches. During the landing phase of the CMJ, knee joint injuries often occur in the ligaments of the knee (Collings et al., 2019). CMJ is also commonly used by sports injury researchers as a non-contact injury screening test for athletes (Read et al., 2018).

Furthermore, CMJ is commonly used to monitor acute neuromuscular readiness and fatigue (Heishman et al., 2019). The decrease in athletic performance associated with running fatigue is partly due to inadequate central nervous system (CNS) drive of motor neurons and poor muscle executive function (Gandevia, 2001). In addition, previous studies have shown that amateur runners have a higher rate of musculoskeletal injury than properly coached runners after running fatigue (Bennell and Crossley, 1996) and most of the injuries occurred in the lower extremities (Araujo et al., 2015). Maclaren and others reported that bone overuse injuries during long distance running may be caused by central nervous fatigue resulting in a weakened ability of muscles to absorb impact (Maclaren, et al., 1989). However, recent research shows that the asymmetry of several gait variables measured before the onset of fatigue change post fatigue in healthy males (Radzak et al., 2017). Previous studies on the symmetry changes caused by motion variability post-fatigue are lacking, however, changes in symmetry during movement may cause non-contact injuries.

The assessment of interlimb symmetry in functional tasks is an important means of injury tendency screening (Kiesel et al., 2007) and sports performance assessment (Lockie et al., 2015). Therefore, a further factor that contributes to poor performance and damage during CMJ tasks is the presence of asymmetry. Posture asymmetry can be defined as the corresponding body limb’s unevenness or mechanical imbalance (Gao et al., 2020b). Increased asymmetry may negatively affect athletic performance (Bishop et al., 2018), the incidence of non-contact injuries (Izovska et al., 2019), and athletic efficiency (Beck et al., 2018). Higher symmetry is significantly associated with better athletic performance, as demonstrated by sprinters (Trivers et al., 2014) and triathletes (Bini and Hume, 2015). Jumping-based tasks are often used as one of the methods to evaluate movement asymmetry. Bishop et al. ‘s vertical jump test study using young female soccer players revealed that higher asymmetrical scores were significantly associated with slower sprint times (Bishop et al., 2018). In addition, studies have reported that 70% of ACL tears occur in non-contact mechanisms, which are caused by the enormous torsional rotation force caused by the rapid deceleration of the individual foot when it hits the ground (Griffin et al., 2006).

Radzak et al. reported that running fatigue weakened the symmetry of joint stiffness at the knee (Radzak et al., 2017). However, the study observed that the symmetry of joint stiffness showed a trend towards asymmetry, though statistical significance was not observed. Symmetry changes caused by running fatigue in healthy individuals have not been adequately studied. Previous studies have reported on the symmetry evaluation of CMJ. Likewise, most of the studies focused on discrete data checks based on extreme values and ignored the symmetry changes in time series data during the propulsion and landing phases. The commonly used inspection methods include symmetry index (SI) (Gao et al., 2020b) and symmetry Angle (SA) (Gao et al., 2020a). The limitations of these assessment methods are that they do not consider the time shift between the left and right legs and do not divide variables into different planes of symmetry. Symmetry Function is a new method for evaluating the symmetry of continuous parameters and was initially proposed by Nigg et al. (2013). The methodology outlined has since been applied in many biomechanical studies (Nigg et al., 2013). The aim of the current study was to compare the knee symmetry changes during CMJ before and after fatigue induced exercise. The objective was to explore the potential mechanism of knee joint injury caused by symmetry changes induced by acute fatigue during a CMJ task. Thus, providing implications for injury prevention and motor skill assessment. Therefore, the current study makes the following three assumptions: 1) Before acute fatigue, the asymmetry of kinematic and dynamic parameters of the knee joint only exists in partial push-off and landing stages, and the degree of asymmetry is not apparent. 2) After acute fatigue, asymmetry of kinematic and dynamic parameters of the knee exists throughout the push-off and landing phases. 3) After fatigue, the joint Angle and joint moment symmetry will deteriorate in all three anatomical planes compared with prior fatigue.

## Methods

### Participants

Twelve male amateur runners with a dominant right leg were recruited in this study. Specific anthropometric information is shown in Table 1. Dominant limbs were defined as kicking a ball leg preference, and amateur runners were defined as running at least two to three times a week for less than 45 min or less than 10 km (Koblbauer et al., 2014). Subjects were injury-free or pain-free for at least 6 months prior to testing and were screened for previous injuries that might affect their lower limbs symmetry. All subjects completed informed consent forms prior to data collection, explaining experimental procedures. The ethics Committee of Ningbo University Research Institute for research using human subjects approved the study (code: RAGH20200529).

**TABLE 1:** Descriptive characteristics of 12 participants.

Information	Mean	Sd
Age (year)	24.92	1.16
Height (cm)	175.67	3.42
Weight (kg)	71.58	4.01
BMI (kg/m^2^)	23.19	0.96

BMI, body mass index.

### Date collection

**FIGURE 1:**
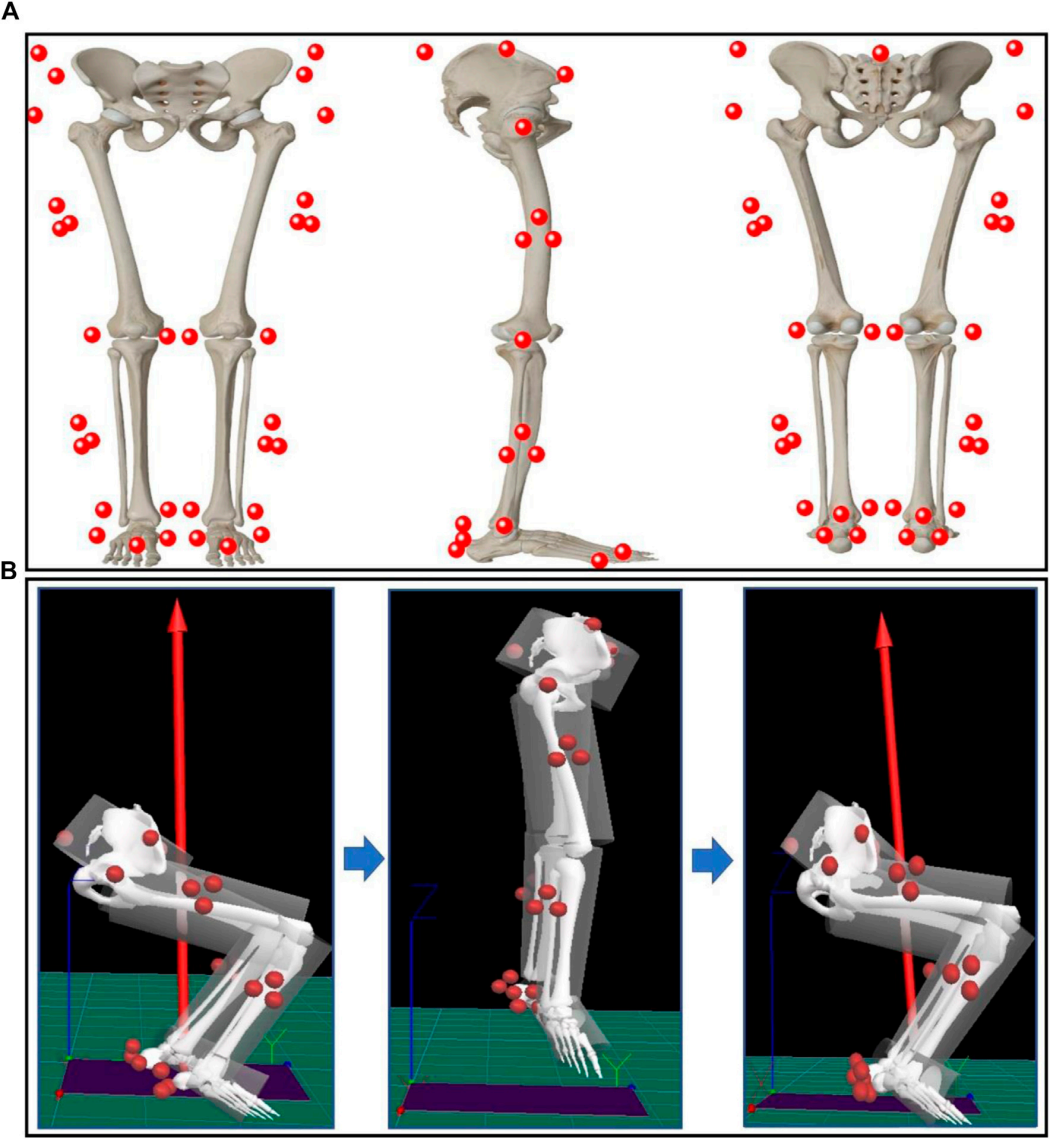
**(A)** Marker-set Placement. **(B)** Over-ground CMJ Test and Biomechanical variable collection.

Three-dimensional kinematic and kinetic data were collected using the Vicon motion capture system (Vicon Metrics Ltd, Oxford, United Kingdom) based on eight high-speed cameras at 200 Hz and an embedded force plate (Kistler, Winterthur, Switzerland) at 1000 Hz. Twenty-six retroreflective markers with a diameter of 14 mm and four marker clusters were attached to the corresponding anatomical positions of the participants’ bilateral lower limbs and pelvis to generate a trajectory that could be captured by the device (Figure 1). The exact markers were bilaterally placed on the distal interphalangeal joint of the second toe, the first and fifth metatarsal heads, medial malleolus and lateral malleolus, medial femoral epicondyles and lateral femoral epicondyles, greater trochanters, anterior superior iliac spines, iliac crests and the joint space between the fifth lumbar and the first sacral spinous, respectively. Furthermore, the six marker clusters were placed on the heel counter of the shoes, mid-thigh and mid-shank of both limbs. A computerized Polar heart rate band (Polar RS100, Polar Electro Oy, Woodbury, NY, United States) and 15-point Borg self-induced fatigue Scale (Rating of Perceived Exertion, RPE, 6–20 Borg’s scale) (Borg, 1998) were used to monitor heart rate and fatigue levels during the prolonged-running protocol.

### Test protocol

The experiment was divided into three stages: 1) Data collection for the CMJ test before acute running fatigue. 2) Acute fatigue experiment induced by running (conducted on a motorized treadmill in the laboratory) and 3) Collection of CMJ test data following acute running fatigue. Data was collected at the same time of day (morning testing) to minimize the data contamination effects of diurnal variation. Participants were instructed to warm up with a 10-min jog after familiarizing themselves with the lab environment and procedures. The CMJ test was then performed until three complete data sets of bilateral lower limbs were collected successfully. Participants were told to jump as high as they could use standard procedures. As outlined by Koblbauer et al., a prolonged-running protocol was applied to induce acute running fatigue (Koblbauer et al., 2014). Participants walked on the treadmill at their initial speed (6 km/h). Then the speed was then increased by 1 km/h every 2 min and the RPE index and heart rate were recorded and stop increasing speed until the RPE index was asked to reach 13 (somewhat hard). The subjects were then asked to run at this speed until fatigued. Fatigue points were defined as running with a real-time heart rate greater than 90% of maximum heart rate or RPE index greater than 17 (very hard) that remained elevated for 2 minutes. Specific implementation details have been reported in previous study (Gao et al., 2020a). We then performed the CMJ test again within 5 min of completion of the fatigue protocol (step 3) (Mei et al., 2019).

### Data processing

The markers trajectory and Vertical ground reaction force data were processed using a two-order Butterworth low-pass filter with cutoff frequencies of 16 and 50 Hz, respectively (Gao et al., 2020a). The determination of the pushing and landing phases has been described in a previous study (Yu et al., 2020). Visual 3D human motion analysis software (v6; C-Motion, Inc, Germantown, MD, United States) was used to perform inverse kinematics and inverse kinetics algorithms to calculate joint angles and joint moment in the sagittal coronal and horizontal planes of the knee joints. The knee joint moment was standardized by individual weight to reduce the error caused by individual characteristics. Formula 1 shows the method of calculating jump height by time of flight (Bosco et al., 1983).

$$\mathrm{jump height}\left(m\right)=\frac{9.80m\cdot s^{-2}\times \;\text{flight time}{\left(s\right)}^{2}}{8}$$ (1)

In order to compare the symmetry of the entire push-off and landing stages of bilateral knee joints, symmetry function was applied to this study (Nigg et al., 2013).

$$\mathrm{SF=}\int_{\mathrm{t=}t_{1}}^{t_{2}}A|x_{r}\left(t\right)-x_{l}\left(t\right)|\mathrm{dt}$$ (2)

$$A=\frac{2}{\mathrm{range}\left(x_{r}\left(t\right)\right)+\mathrm{range}\left(x_{l}\left(t\right)\right)}$$ (3)

Where $x_{r}\left(t\right)$ was defined as the value of the parameter recorded for the right knee at the time t. $x_{l}\left(t\right)$ was defined as the value of the parameter recorded for the left knee at the time t SF is the symmetry function. In addition, $t_{1}$ and $t_{2}$ stand for the time at heel strike and time at take-off, respectively. Formula 2 is an integrand and is referred to as the SF. The time-dependent information of symmetry at 101-time points in the action stage is reflected in the SF. The closer SF is to 0, the more symmetric the bilateral variables are defined (Formula 3). This study set a ± 5% symmetry threshold to discriminate asymmetry areas (Winiarski et al., 2021).

### Statistical analysis

The data of time series attributes were standardized to 101 data points using a linear interpolation method to facilitate statistical comparisons (Holden et al., 1997). SF was used to compare the joint angles and shutdown moment of bilateral knees throughout the push-off and landing phases. This check was performed pre-fatigue and post-fatigue, respectively. The Shapiro-Wilks test was used to verify the normality of the data using SPSS (Version 19; SPSS, Inc, Chicago, IL, United States). A paired sample *t*-test algorithm package within the open-source data analyst Statistical Parametric Mapping (SPM) was used to compare SF changes before and after fatigue in MATLAB (Version: R2019a, The MathWorks, Natick, MA, United States). Paired sample *t*-test was used to check the difference of jump height before and after running-induced fatigue protocol in SPSS software (version 26, SPSS, Chicago, IL, United States). The significance level was set to 0.05.

## Results

### Pre-fatigue biomechanical variables

**FIGURE 2:**
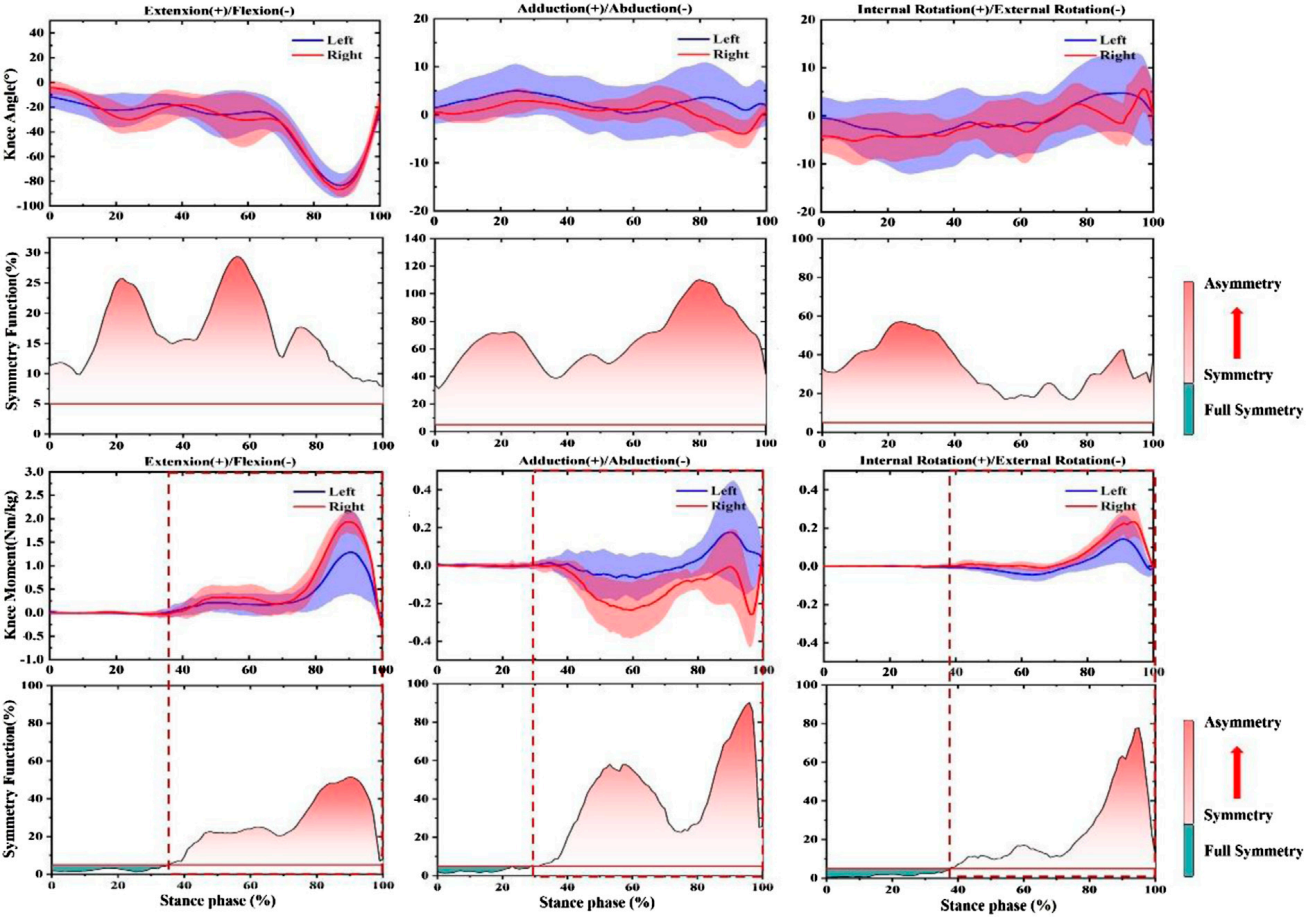
Illustration of SF degree in bilateral knee joint angle and moment during push-off stage before prolonged-running protocol for the CMJ test. Note: The red fill represents the degree of asymmetry, and the darker the color, the more asymmetry. The green fill represents full symmetry.

In the push-off phase before fatigue, SF of knee joint Angle and moment were both more significant than the symmetry threshold (SF > 0.05), as shown in Figure 2. More specifically, in the knee angle’s sagittal view, 18–25% and 45–60% of the support period showed larger SF. Moreover, 70–90% and 20–30% of the support period of SF reached the highest in the coronal and horizontal planes, respectively. The trend of knee joint moment. 0–35%, 0–30%, and 0–38% of SF in the stance period were lower than the symmetry threshold (SF < 0.05), respectively. In addition, SF increased sharply from 70% in the support period until 90% and reached a peak of 0.5 in the sagittal plane. 50–60% and 90–95% showed larger SF in the coronal plane. SF increased sharply from 38% until 95% reached a peak of 0.8 in the horizontal plane.

**FIGURE 3:**
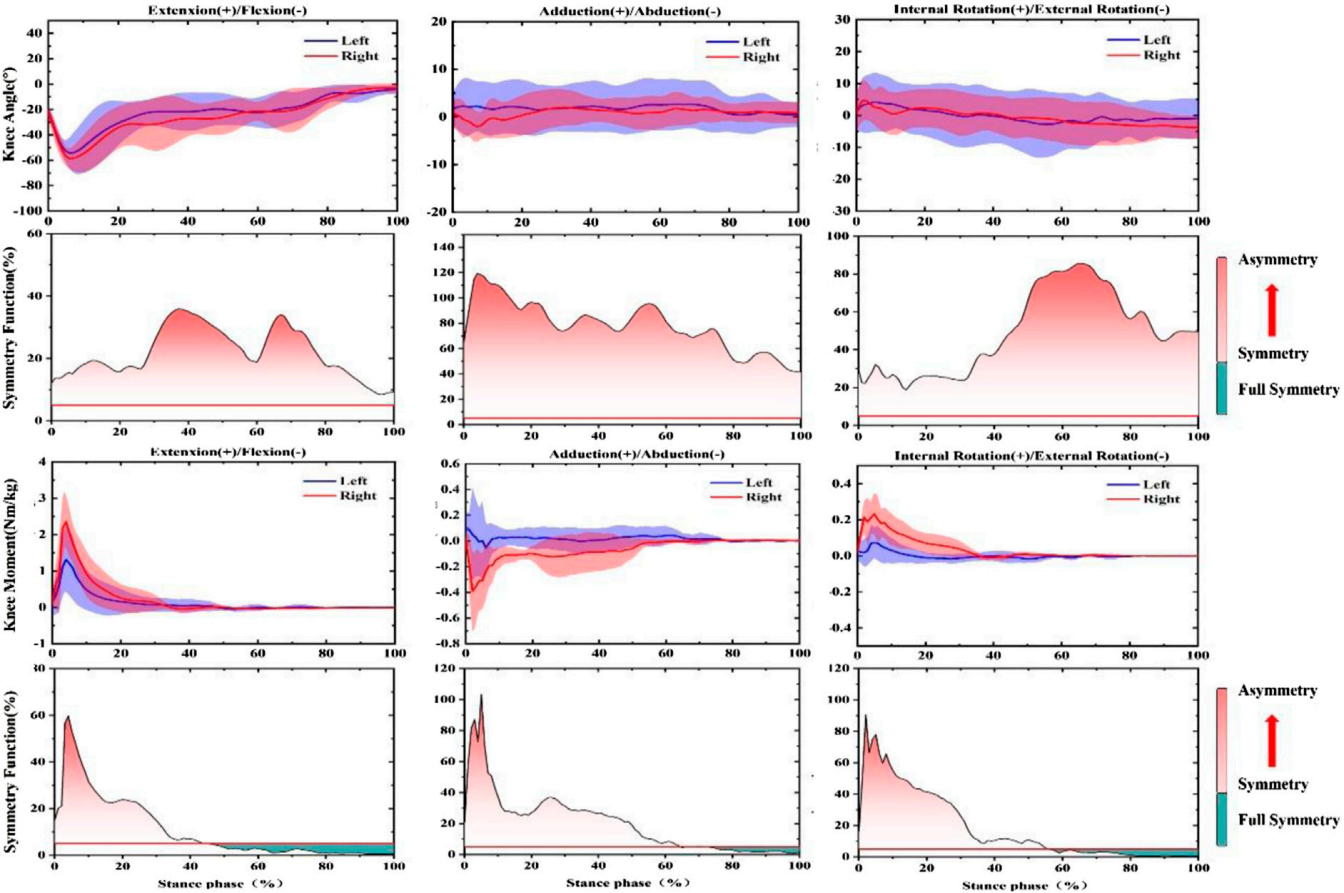
Illustration of SF degree in bilateral knee joint angle and moment during the landing phase before prolonged-running protocol for the CMJ test. Note: The red fill represents the degree of asymmetry, and the darker the color, the more asymmetry. The green fill represents full symmetry.

According to the results in Figure 3, SF of knee joint Angle and moment were both greater than the symmetry threshold on three anatomical planes (SF > 0.05). 35–45% and 65–70% of SF in the knee angle of the support period showed larger SF in the sagittal plane. SF peaked at 120% at 5% at the coronal level and gradually decreased to 0.5. SF increased sharply from 30% in the support period until 65% reached a peak of 0.85 in the horizontal plane. In addition, the trend of the knee joint moment. 42–100%, 72–100%, and 53–100% of SF during the stance period were lower than the symmetry threshold (SF < 0.05), respectively. The SF of knee moment in 5% reached peak values at 0.6 and 0.9, and then followed a downward trend in the sagittal and coronal planes. In the horizontal plane, 3% arrived a peak at 0.9 and then it also followed a downward trend.

### Post-fatigued biomechanical variables

**FIGURE 4:**
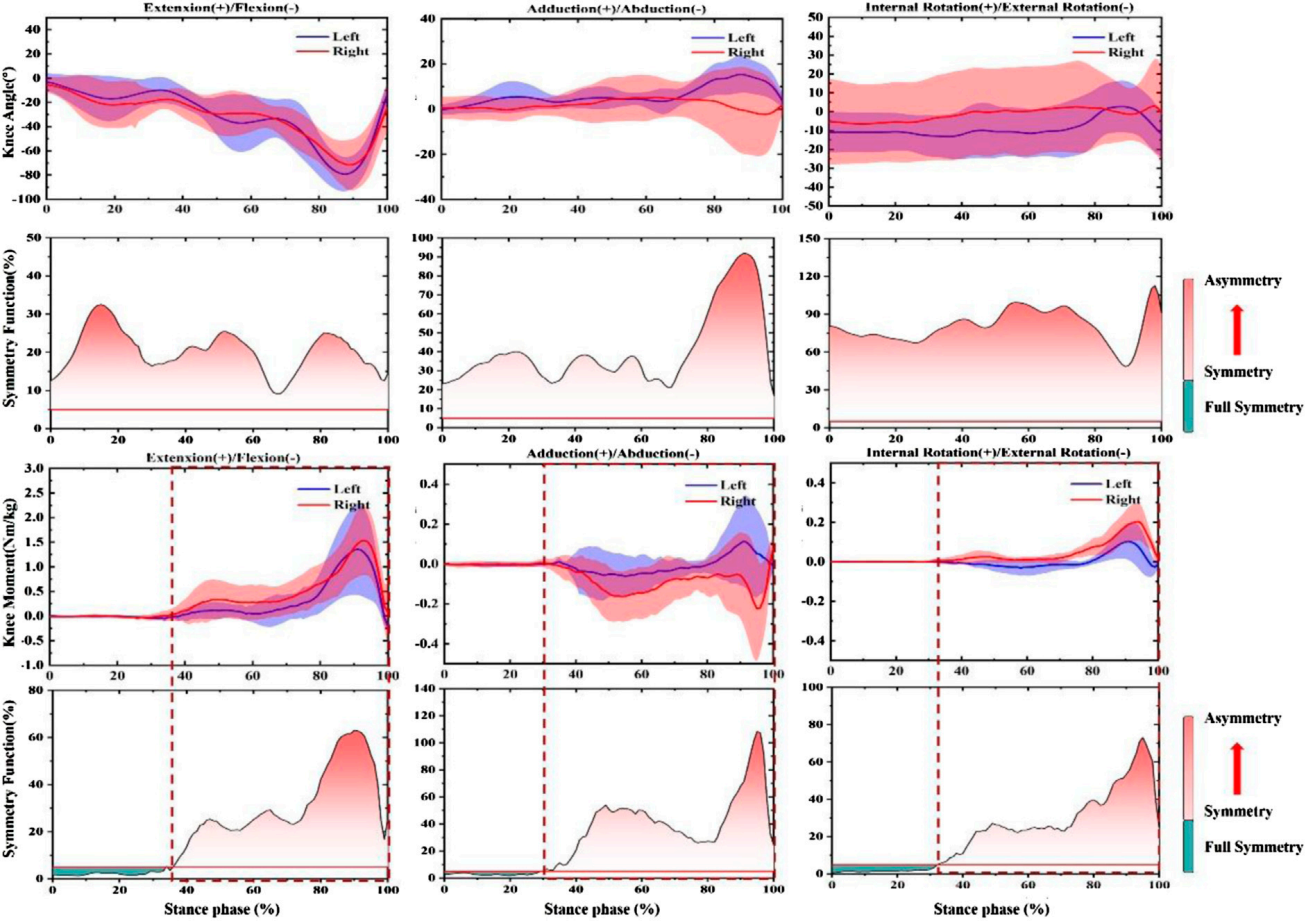
Illustration of SF degree in bilateral knee joint angle and moment during push-off phase after prolonged-running protocol for the CMJ test. Note: The red fill represents the degree of asymmetry, and the darker the color, the more asymmetry. The green fill represents full symmetry.

In the push-off phase after fatigue, SF of the knee joint Angle and moment were both greater than the symmetry threshold (SF > 0.05), as shown in Figure 4. More specifically, in the knee angle’s sagittal plane, 10–20% of the support period showed larger SF. In addition, 85–95%, was the largest SF in the coronal plane. SF exhibits great asymmetry in the horizontal plane throughout the stance phase (SF > 0.5). Moreover, 0–33%, 0–30%, and 0–34% of SF recorded for the knee joint moment of the support period were lower than the symmetry threshold (SF < 0.05), respectively. In addition, 85–95% and 90–95% of the support period of SF reached the highest values in the sagittal and coronal planes, respectively. The horizontal plane, which is 95%, recorded a peak of 0.7, then followed a downward trend.

**FIGURE 5:**
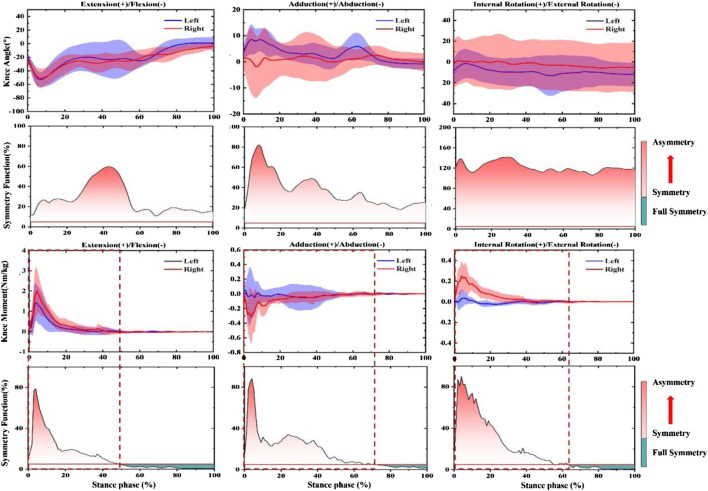
Illustration of SF degree in bilateral knee joint angle and moment during landing phase after prolonged-running protocol for the CMJ test. Note: The red fill represents the degree of asymmetry, and the darker the color, the more asymmetry. The green fill represents full symmetry.

According to the results in Figure 5, SF for knee joint angle and moment both were greater than the symmetry threshold in three anatomical planes (SF > 0.05).30–50% and 10–30% of SF of knee angle during the support period showed larger SF in the sagittal plane and coronal plane. SF exhibits great asymmetry throughout the stance phase (SF > 0.9). In addition, from the trend of knee joint moment. 50–100%, 70–100%, and 62–100% of SF of the stance period were lower than the symmetry threshold (SF < 0.05), respectively. The SF of the knee moment at 5% arrived at peak values at 0.8, 0.9, and 0.9, then it observed a downward trend in the sagittal plane, coronal plane, and horizontal plane, respectively.

### Jump performance

The jumping heights before and after the running-induced Fatigue protocol were 275.2 (42.1) mm and 268.5 (28.3) mm, respectively. No significant difference in jump height was observed between these two states (*p* = 0.39).

### Symmetry function

**FIGURE 6:**
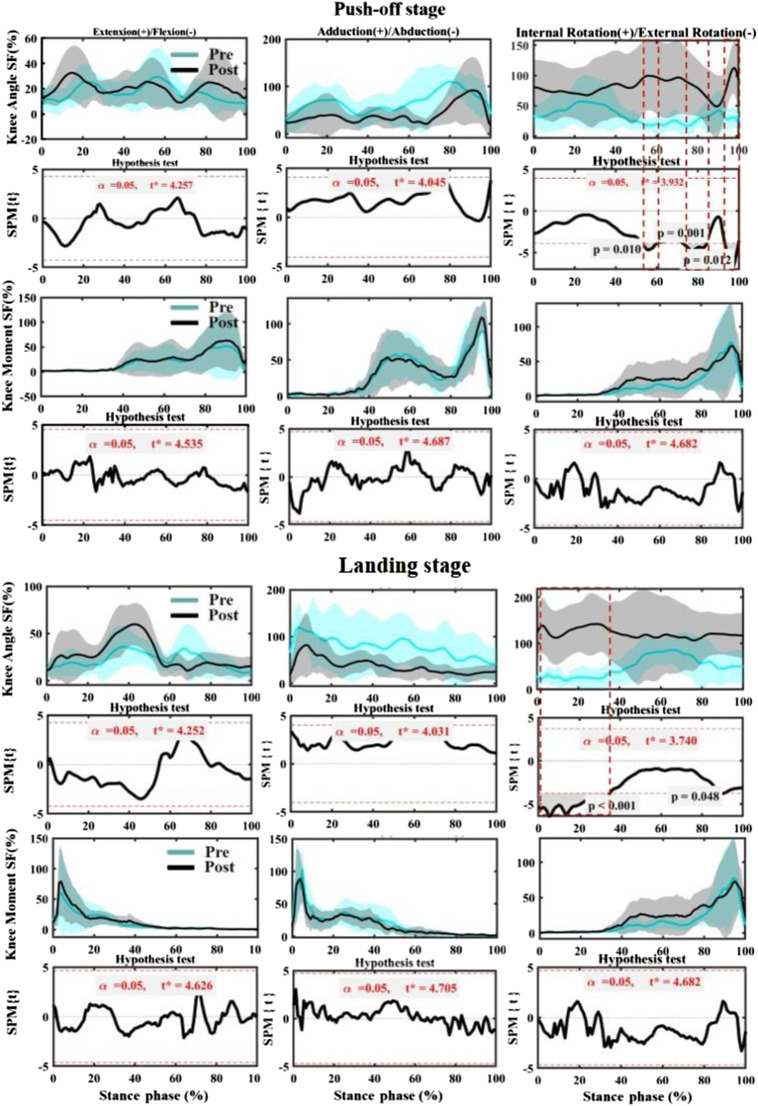
Comparing the mean values of SF of knee angle and moment from all participants between fatigues (pre-fatigue; post fatigue). Note: the dotted boxes indicate a statistically significant difference, *p* < 0.05. Pre means Pre-fatigue, and Post means post-fatigue.

As shown in Figure 6, no significant differences in knee Angle SF were observed in the push-off phase between pre-fatigue and post-fatigue in the sagittal plane and coronal plane (*p* > 0.05). However, the SF of post-fatigue was significantly higher than pre-fatigue during the push-off stage (52–60%, 75–85%, and 92–100%). Moreover, after fatigue, a significant increase in SF occurred in the landing phase of 0–35% (*p* < 0.05). The knee moment did not change significantly in all stages and planes.

## Discussion

This study aimed to examine the symmetry changes of continuous biomechanical parameters of bilateral knee joints during the CMJ task before and after fatigue. Considering the time shift between the left limb and right limb, SF was used to evaluate symmetry in this study (Nigg et al., 2013). The results of this study are consistent with hypothesis 2), but, interestingly, not wholly consistent with the hypothesis 1) and hypothesis 3). Specifically, the angles and moment of bilateral knee joints were asymmetrical in all three anatomical planes before fatigue. The RPE>17 and the HR>90%HRmax during the prolonged-running protocol were observed in this study, suggesting that fatigue exists in participants even there was no significant difference in CMJ performance, the results may be inconsistent with previous studies due to different intervention methods (Gandevia, 2001). Therefore, in future studies, we should add more objective indicators to monitor and quantitative neuromuscular fatigue (e.g., neuromuscular electrical stimulation and IMVC (isometric maximal voluntary contraction). In addition, acute neuromuscular fatigue only caused deterioration of external rotation symmetry of the knee at push-off and landing stage but did not significantly affect other parameters. A previous study reports that the acceleration of body mass needs to be as high as possible during a bilateral leg stretch and is an essential factor in CMJ performance (Cormie et al., 2007). Reverse movement vertical jump task requires high mechanical output and coordination modes of the individual performing the task, and the individual needs to push the body upward at the highest possible speed (Bobbert and van Ingen Schenau, 1988). Therefore, the difference in the explosive force of bilateral lower limb muscles may cause the asymmetry of bilateral knee joints during the push-off stage. Previous studies have shown that the left limb usually plays a stabilizing role in the performance of motor tasks on both sides (Sadeghi et al., 2000). The left knee showed greater angle of motion during periods of high asymmetry (18–25% and 45–60% in sagittal, 79–90% in coronal, and 20–30% in horizontal). Probably to create more stability for the push-off phase. Propulsive force is mainly related to leg muscle power generation, while support and control function is mainly related to limb power absorption behaviour (Sadeghi et al., 1997). Similarly, the right knee joint showed more joint moment in the coronal plane and horizontal plane, which may be a potential mechanism by which the dominant limb mainly performs propulsive functions (Sadeghi et al., 2000). Likewise, the larger adduction moment of the left knee joint may result in greater load on the medial side of the knee joint of the non-dominant limb. (Kastelein et al., 2008). The complete symmetry of the knee moment only appeared during reverse pre-stretching of the CMJ task, suggesting that the movement is most balanced at this stage. The skeletal muscles of the lower limbs in the landing phase need the force to be generated rapidly during eccentric braking to cushion the joint load (Laffaye and Wagner, 2013). The higher flexion angle of the right knee observed in the sagittal plane during the landing stage means that the right knee bears more buffering work during the CMJ task. Previous studies have shown that dominant limbs have more muscle strength and task attributes, which is consistent with the results of this study (Sadeghi et al., 2000).

Reverse pre-stretching prior to muscle contraction puts the muscle at a higher level of activity, resulting in a greater joint moment during the push-up phase of the CMJ task (Bobbert et al., 1996; Schenau et al., 1997). This phenomenon may be related to reverse motion allowing elastic potential energy to be stored and reused in skeletal muscles and tendons for better performance (Anderson and Pandy, 1993). The right knee showed a greater flexion angle and extension moment in the push-off phase after the fatigue protocol was implemented, which was consistent with the performance before fatigue, suggesting that fatigue did not affect the contribution of the sagittal plane of both knees during propulsion (Echeverria et al., 2010). However, the left knee joint was observed to have a large external rotation angle throughout the stage, which may be caused by reduced neuromuscular control due to loss of muscle activation capacity (Gandevia, 2001), but further research is needed to verify if kinematics measures could be used to infer neuromuscular control. Meanwhile, the right knee joint’s greater abduction rotation moment may be associated with arch collapse after fatigue, which is a potential factor for excessive contact force on the medial tibia (Mei et al., 2019). The knee of the right leg showed more flexion angles after fatigue than the opposite leg, suggesting that the right lower limb made a greater contribution to buffering during the landing phase. Meanwhile, the higher extension moment of the right knee suggested a greater sagittal load on the knee of the dominant limb after fatigue. Noticeably, more knee external rotation occurred in the left limb, and the SF degree of the bilateral limb exceeded 100%, which may be due to the movement variability caused by the weak fatigue tolerance threshold in the non-dominant limb. Whether this variability causes excessive meniscus load in the left knee joint should be considered in future studies (William Abraham, 2020). Additionally, the large internal rotation moment observed in the right knee joint may be a protective mechanism for improving joint stiffness (Brughelli and Cronin, 2008).

Fatigue may deteriorate the symmetry of both limbs, with one limb at greater risk of non-contact injury. The symmetry of bilateral knee joints after fatigue is significantly less than before (Smeets et al., 2019; Xiang et al., 2022). Although our study has not evaluated injury individuals or injury risk, the current study results suggest that asymmetry magnitude in parameters previously associated with non-contact knee joint injury may be fatigue susceptible (Smeets et al., 2019). In the current study, the asymmetry changes of the knee angle in the horizontal plane after fatigue can support this view. In the push-off stage, more significant asymmetry appears after fatigue. This asymmetry may be caused by the body changing the distribution of tasks in both lower limbs. Therefore, the whole body is more stable after fatigue while maintaining performance. However, the knee joint moment did not change in any of the three anatomical planes during the push-off stage. This finding can potentially explain that the change in kinematic symmetry is benign and does not cause excessive load on the knee joint. Interestingly, this finding contradicts previous research suggesting that fatigue causes lower limb kinematics to become more symmetrical (Gao et al., 2020a). Different motion patterns and the examination of the symmetry of the whole time series may be the main reasons for the different conclusions. The same phenomenon is observed in the landing stage when the forefoot contacts the ground, which is caused by excessive external rotation of the knee joint of the non-dominant limb. Previous studies have reported that the hemimembrane muscle plays a limiting role in external rotation during knee flexion (Robinson et al., 2004). Future studies should consider the signal measurements of hemimembrane muscle of non-dominant limb to better explain this phenomenon. In addition, forced external rotation during knee flexion has been previously linked to an avulsion fracture at the tibial straight arm insertion of the tendon (Chan et al., 1999; Tao et al., 2021; Xu et al., 2022). Kinematics changes of asymmetry after fatigue found within the healthy individuals of this study warrant further prospective evaluations exploring fatigue and knee asymmetry development in the CMJ.

There are some limitations in this study. Firstly, all the subjects recruited in this study were amateur male runners, and future studies should also consider the differences that may occur in different levels, such as elite runners. Secondly, the current study only focused on the biomechanical symmetry changes of the knee joint, and future studies should analyze overall lower limb symmetry. Single angle and moment parameters cannot fully explain the load and work done by the knee joint. Future studies should conduct more detailed analyses based on more relevant parameters such as joint power, mechanics, muscular activity and contact forces. In addition, only male amateur runners were included in this study, and gender-induced symmetry differences will be considered in future studies. In the end, the validity of the findings may be compromised by potential errors of the subjective quantification of neuromuscular fatigue (e.g., RPE>17). Thus, these comparisons are preliminary. Further studies using objective neuromuscular fatigue indicators such as neuromuscular electrical stimulation and IMVC may provide a different assessment of the quantitation of running fatigue.

## Conclusion

This study investigated the changes in knee joint symmetry of angle and moment during a CMJ task before and after running-induced fatigue in 12 male recreational runners. SF was used to check the symmetry of the time series parameters of the whole motion cycle. The findings from the study were that there were different degrees of asymmetry in the push-off and landing stages of both knee joints before and after fatigue. The symmetry of the external rotation angle of the knee joint deteriorated after fatigue during the propulsive period during the push-off stage and the forefoot landing period in the landing stage. The change was mainly caused by excessive external rotation of the dominant knee joint. This finding may provide evidence for the asymmetry of knee joints caused by fatigue during jumping tasks. Future studies are needed to confirem the potential relationships between knee joints asymmetry and fatigue and to investigate the possible relationship to jump-related injuries and knee asymmetry.

## Data Availability

The original contributions presented in the study are included in the article/Supplementary Materials, further inquiries can be directed to the corresponding authors.
